# Adenosine triphosphate-binding cassette transporters: key players in maintaining fetal health during pregnancy

**DOI:** 10.3389/fendo.2026.1858727

**Published:** 2026-07-16

**Authors:** Xian-Hong Wang, Ghazala Saeed, Ali Afzal, Umair Ali Khan Saddozai, Gui-Min Shao, Amna Rehman, Syeda Eisha Hamid, Si-Ying Pan, Xin-Ying Ji, Muhammad Babar Khawar

**Affiliations:** 1 Department of Internal Medicine and Pediatrics, Medical School, Henan Provincial Research Center of Engineering Technology for Nuclear Protein Medical Detection, Zhengzhou Health College, Zhengzhou, Henan, China; 2 Cell & Molecular Biology Lab, Institute of Zoology, University of the Punjab, Lahore, Pakistan; 3 Shenzhen Institutes of Advanced Technology, Chinese Academy of Sciences, Shenzhen, China; 4 University of Chinese Academy of Sciences, Beijing, China; 5 School of Basic Medical Sciences & School of Public Health, Faculty of Medicine, Yangzhou University., Yangzhou, China; 6 Department of Science and Technology, Jinzhong College of Health, Jinzhong, Shanxi, China; 7 Department of Public Health, Shinawatra University., Bangkok, Thailand; 8 School of Life Sciences and Biotechnology, Shanghai Jiao Tong University, Shanghai, China; 9 Department of Obstetrics and Gynecology, Medical School, Henan Provincial Research Center of Engineering Technology for Nuclear Protein Medical Detection, Zhengzhou Health College, Zhengzhou, Henan, China; 10 Department of Microbiology and Immunology, Medical School, Henan Provincial Research Center of Engineering and Technology for Medical Detection of Nuclear Protein, Zhengzhou Health College, Zhengzhou, Henan, China

**Keywords:** ATP-binding cassette transporters, fetal health, placenta, preeclampsia, pregnancy, preterm birth

## Abstract

ATP binding cassette (ABC) transporters are active efflux proteins that move substrates against the gradient via hydrolysis of ATP, and regulate fetal metabolic homeostasis and chemical exposure. The maternal-fetal interface represents a barrier for key transporters involved in xenobiotic efflux, reverse cholesterol transport, bile acid handling, metabolism of steroid hormones and immune tolerance. We combine recent data from cryo-electron microscopy-based structure with recent single-cell transcriptomic data and *ex vivo* studies of placenta pharmacology to explore the underlying mechanism of transporter dysfunction in preeclampsia, intrahepatic cholestasis of pregnancy, preterm birth, gestational diabetes mellitus, intrauterine growth restriction, and early miscarriage. NRF2-dependent antioxidant signaling, HIF-1α-dependent suppression of BCRP under hypoxia, cytokine-dependent downregulation by TNF-α and IL-1β, and autophagy. Key translation opportunities in clinical pharmacogenomics are highlighted, such as near-term ABCG2 Q141K genotyping for glyburide prescribing in gestational diabetes, and ABCB4 variant profiling for cholestasis risk.

## 1 Introduction

During pregnancy, the body undergoes profound physiological adaptation, particularly in the formation of the placenta. It faces competing demands as it must transport nutrients and endocrine signals to the fetus while blocking xenobiotics, oxidized lipids, and harmful maternal metabolites (1, 2). One of the key players in such processes are a group of active efflux proteins known as ATP binding cassette (ABC) transporters, whose mechanism involves transport of various substances against their concentration gradient using the energy provided by ATP (3, 4).

Currently, there are 48 ABC genes divided into 7 different subfamilies (ABCA-ABCG), based on phylogenetic relationships, domain organization, and amino acid sequence homology (4). Among these, 44 ABC genes encode for membrane transporters (4). ABC transporters have a common architecture in which two transmembrane domains (TMDs) are responsible for specificity and transportation, whereas, two nucleotide-binding domains (NBDs) ensure ATP binding and catalyze its hydrolysis to produce large scale conformational movements that results in substance transportation across the phospholipid bilayer (5, 6). Recent atomic resolution cryo-EM structures of representatives of the 5 transporting subfamilies (ABCA-ABCD, and ABCG) have shown that while the structure of NBDs remains conserved, TMD architecture varies drastically leading to the wide physiological substrates including ions, lipids, bile acids, steroid hormones, glutathione conjugates, and various xenobiotics (4, 7).

Among ABC transporters involved in the physiology of the placenta, there are primarily P-gp/ABCB1 and BCRP/ABCG2 that are expressed in the apical (facing the maternal environment) brush-border membrane of the syncytiotrophoblast, thereby preventing passage of the maternal compounds into the fetal circulation (1, 8). Besides, transporters from the ABCA, ABCC, and ABCG subfamilies have an important role in maintenance of placental sterols balance, bile acids transport, steroid hormone metabolism, and immunological processes (9, 10). Notably, the regulation of these transporters is a dynamic process that depends on hormonal and other factors which makes them susceptible to perturbation in case of obstetric complications (1, 11).

The importance of the involvement of ABC transporters in obstetric pathology is reflected in their significant prevalence of associated diseases. For instance, preeclampsia affects 2-8% of pregnancies and causes approximately 76,000 maternal deaths and 500,000 fetal deaths annually (12, 13). Premature birth occurs in 9.5-11% of deliveries globally (14). Gestational diabetes mellitus (GDM) affects 14% of pregnancies (15), and spontaneous abortion occurs in up to 10-20% of pregnancies (16). Despite the epidemiological weight of these conditions, the molecular mechanisms by which ABC transporter dysfunction contributes to their pathogenesis remain incompletely understood.

Existing reviews on placental ABC transporters largely focus on subfamilies ABCB, ABCC, and ABCG and their pharmaceutical relevance, without integrating recent cryo-EM structural advances, and full spectrum of obstetric complications across seven subfamilies (8, 17, 18). We, herein, provide an evidence-based synthesis on how the placental ABC transporters maintain feto-placental homeostasis and how their dysfunction contributes to preeclampsia, preterm birth, intrahepatic cholestasis of pregnancy (ICP), GDM, intrauterine growth restriction, and miscarriage. Our review distinguishes itself from prior work in three key aspects. First, it systematically covers all seven ABC transporter subfamilies with full spectrum of obstetric complications. Second, it employs a cell type-resolved expression framework that maps each transporter to the syncytiotrophoblast, cytotrophoblast, and extravillous trophoblast compartments across gestational stages. Third, it integrates recent single-cell transcriptomic and multi-omics data with *ex vivo* placental pharmacology to generate clinical pharmacogenomics implications.

## 2 Biology of ABC transporter subfamilies

**Figure 1:**
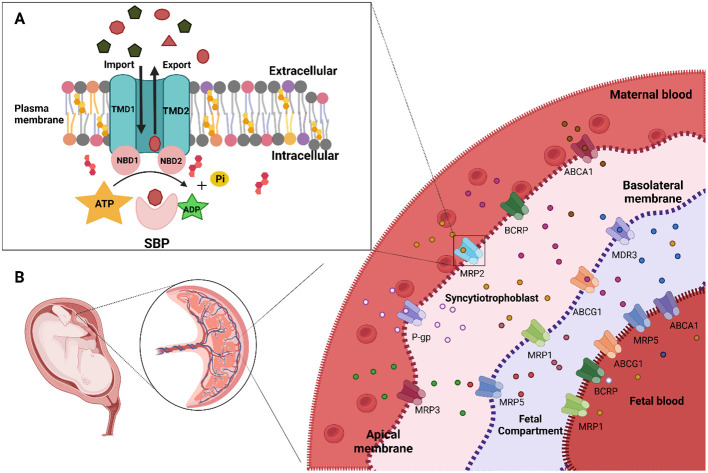
Configuration and locality of ABC transporters in the placenta. **(A)** The basic structure of the ABC transporter consists of two TMD domains and two NBD domains; TMDs make translocation pathways and NBDs provide energy for translocation by hydrolyzing ATP. Any substrate that passes by the transporter either inside or outside of the cell binds to a substrate-binding protein involved in substrate specificity. **(B)** Illustration of the cross-section of the placenta and highlights some ABC transporters present in different locations and performing their specific functions by transporting different substances across membranes.

Among the seven subfamilies of human ABC transporters, ABCA, ABCB, ABCC, ABCD, and ABCG encode membrane-spanning transporter proteins, while ABCE and ABCF encode soluble proteins lacking TMDs that participate in ribosome recycling and translation regulation (19, 20) (summarized in **Table 1**). The ABCA subfamily, for instance, distinguishes itself through a unique architecture featuring large extracellular domains and cytoplasmic regulatory elements that facilitate the transport of complex lipids. Conversely, the ABCD1–3 functions primarily as peroxisomal half-transporters, which assemble into homodimers to govern the intracellular metabolism of fatty acids and cobalamin (26). These transporters exemplify the type-IV-fold, characterized by a two-fold symmetric arrangement where specific transmembrane helices are domain-swapped to enable alternating inward- and outward-facing conformations. The canonical transporter architecture consists of two TMDs and two NBDs arranged in either a full transporter (one polypeptide chain encoding both TMD–NBD cassettes) or a half-transporter (one TMD–NBD cassette that dimerizes with a partner) configuration (5, 7) (**Figure 1**). The diverse architecture of these domains is fundamentally categorized into several distinct structural folds, which are largely determined by the specific organization and orientation of the transmembrane helices (27). The NBDs function as the engine of the transporter, housing highly conserved motifs such as the Walker A, Walker B, and the ABC signature motif required for the binding and hydrolysis of ATP (28). These cytosolic domains are structurally conserved across the entire superfamily, serving as the universal energy transducer that couples chemical energy to the conformational transitions of the variable TMDs (29, 30).

**Table 1:** ABC transporter subfamilies and associated diseases.

Subfamily	Gene count	Structural features	Key members & substrates	Disease associations	References
ABCA	13	Full transporter (2 TMDs + 2 NBDs); ~2,500 aa; largest human ABC transporters	ABCA1 (cholesterol, phospholipids); ABCA4 (retinal/phosphatidylethanolamine); ABCA7 (lipids, phagocytosis); ABCA12 (lipids, skin barrier)	Tangier disease (ABCA1), Stargardt macular dystrophy (ABCA4), Alzheimer’s risk (ABCA7), harlequin ichthyosis (ABCA12), preeclampsia, IUGR, miscarriage	(4, 7, 21)
ABCB	11	Both full transporters and half-transporters; P-gp (ABCB1) canonical MDR pump; ABCB4 is a phospholipid flippase	ABCB1/P-gp (drugs, xenobiotics, steroids); ABCB4/MDR3 (phosphatidylcholine); ABCB11/BSEP (bile salts); ABCB6 (porphyrins)	Multidrug resistance, ICP (ABCB4/ABCB11), PFIC type 2 (ABCB11), preterm birth, miscarriage, malaria in pregnancy	(4, 22, 23)
ABCC	13	Full transporters; some contain an additional TMD0; encode MRP proteins; CFTR (ABCC7) is an ion channel	ABCC1/MRP1 (GSH conjugates, organic anions); ABCC2/MRP2 (bilirubin, conjugates); ABCC3/MRP3 (bile acids); ABCC4-5 (nucleotides, prostaglandins); ABCC7/CFTR (Cl– ion)	Cystic fibrosis (ABCC7), Dubin-Johnson syndrome (ABCC2), ICP, preeclampsia, GDM, malaria in pregnancy	(7, 11, 24)
ABCD	4	Half-transporters; localized to peroxisomal membrane; function as heterodimers with 2 NBDs	ABCD1 (fatty acids, acyl-CoA); ABCD2 (very long chain fatty acids); ABCD3 (bile acids to peroxisome)	X-linked adrenoleukodystrophy (ABCD1); ABCD3 deficiency / congenital bile acid synthesis disorder; ABCD4-related cobalamin metabolism disorder	(4, 7)
ABCE	1 (ABCE1)	Soluble protein; lacks TMDs; contains 2 NBDs; Fe-S cluster bridging the two NBDs	RNase L inhibitor; ribosome recycling; translation regulation	Involved in antiviral responses; cancer (overexpressed); implicated in placental immune defense	(19, 20)
ABCF	3	Soluble proteins; lack TMDs; contain 2 NBDs separated by linker	Ribosome-associated functions; translation regulation (ABCF1, ABCF2, ABCF3)	Associated with autoimmune conditions; ABCF1 linked to inflammation/immune regulation; reproductive relevance remains emerging	(4)
ABCG	5 (ABCG1–5 + 8)	Half-transporters; function as homodimers or heterodimers; ‘reverse’ architecture (NBD-TMD)	ABCG1 (cholesterol, PC lipids); ABCG2/BCRP (xenobiotics, bile acids, drugs); ABCG5/G8 heterodimer (plant sterols, cholesterol); ABCG4 (cholesterol)	Sitosterolemia (ABCG5/G8), preeclampsia, IUGR, GDM, miscarriage, malaria (ABCG1/G2)	(1, 9, 10, 25)

Abbreviations: aa, amino acids; ABC, ATP-binding cassette; BSEP, bile salt export pump; CFTR, cystic fibrosis transmembrane conductance regulator; GDM, gestational diabetes mellitus; ICP, intrahepatic cholestasis of pregnancy; IUGR, intrauterine growth restriction; MDR, multidrug resistance; MRP, multidrug resistance-associated protein; NBD, nucleotide-binding domain; P-gp, P-glycoprotein; RNase L, ribonuclease L; TMD, transmembrane domain.

Cryo-EM has revolutionized structural understanding of ABC transporters. Multiple structures of ABCB1 (P-gp), ABCG2 (BCRP), and ABCC1 (MRP1) have now been resolved in different substrate-bound, inhibitor-bound, and apo-like states, collectively illuminating the key steps of the transport cycle and providing templates for structure-based drug design (4, 7). The members of the ABCA family form the largest ABC proteins (ABCA1 is around 2,500 amino acids long) because they possess extra regulatory regions associated with lipid transport and, for ABCA1, interaction with acceptor binding, such as ApoA-I (4). The ABCG family forms a unique NBD-TMD topology compared with other exporter proteins and mostly functions as homo- (ABCG1 and ABCG2) or obligatory heterodimers (ABCG5/ABCG8) (10). These advancements have provided high-resolution snapshots of various conformational states, enabling a deeper understanding of the molecular transitions that drive substrate translocation across the lipid bilayer.

Recent research utilizing lipid nanodiscs has further revealed that these conformational transitions are frequently modulated by lipid-competition mechanisms, which actively facilitate substrate release by altering drug-transporter interactions within the vestibule (31). Furthermore, the integration of these structural insights with single-particle analysis under turnover conditions has demonstrated that transporters like ABCG2 can adopt distinct conformational populations to specifically differentiate between substrates and inhibitors (32). Additionally, studies on members like ABCA4 have elucidated how specific amino acid residues coordinate substrate recognition within the TMDs, providing a clearer mechanistic view of how these large transporters process complex lipid substrates. These structural data collectively indicate that the asymmetric movement of subunits, driven by ATP binding and hydrolysis, acts as the primary mechanical force for the translocation process (33).

Furthermore, structural characterization has clarified how pathogenic mutations within conserved motifs of ABCA transporters disrupt these mechanical transitions or substrate recognition, directly linking such functional impairments to severe hereditary pathologies (34, 35). These molecular insights into transporter-pathology relationships underscore the broader physiological significance of ABC proteins, whose dysfunction is implicated in numerous disease pathways resulting from impaired cellular homeostasis (4). Beyond these pathological implications, the discovery of potential import characteristics in certain eukaryotic transporters challenges the long-standing dogma that these proteins function exclusively as exporters (27). For instance, the ABCD subfamily of half-transporters exemplifies functional diversity, primarily operating within peroxisomal membranes to facilitate the trafficking of acyl-CoA esters and cobalamin (26). Specifically, these proteins undergo a cooperative conformational switch where ATP-dependent NBD dimerization triggers an outward-facing state, forcing the substrate-binding cavity to collapse and facilitate translocation into the peroxisomal matrix (36). These structural findings suggest that the specificity of substrate uptake is governed by unique hydrophobic pockets, which accommodate distinct fatty acid chains through precise residue arrangements within the TMDs (37). Moreover, comparisons between the conserved TMD architectures of these half-transporters and their full-length counterparts reveal that the diversification of these translocation pathways has been essential for the evolutionary adaptation to diverse cellular niches (30, 38). Interestingly, recent studies on the BacA mycobacterial transporter reveal that its large internal cavity utilizes a non-canonical, high-volume architecture that facilitates the binding of diverse substrates without relying on high-affinity sites, suggesting a departure from the strict specificity observed in other ABC family members (29). This variation in structural logic suggests that the superfamily’s functional diversity arises from distinct evolutionary pathways, reflecting a broader division into subfamilies defined by unique transmembrane domain sequences and phylogenetic origins (39).

Drug pharmacokinetics depends substantially on ABC transporters expressed in intestine, liver, kidney, blood–brain barrier, and placenta, where they determine bioavailability, volume of distribution, and clearance (40, 41). Clinically, co-administered drugs competing for the same ABC transporter, such as P-gp (ABCB1) and BCRP (ABCG2), can produce significant pharmacokinetic drug-drug interactions which can alter systemic drug exposure and tissue penetration (42). At least 21 ABC genes are causally linked to rare monogenic diseases when mutated, including cystic fibrosis (ABCC7/CFTR), X-linked adrenoleukodystrophy (ABCD1), Tangier disease (ABCA1), Stargardt macular dystrophy (ABCA4), and multiple forms of progressive familial ICP (7). The proof-of-concept provided by CFTR modulators (e.g., elexacaftor–tezacaftor–ivacaftor) demonstrating pharmacological rescue of ABC transporter dysfunction has opened a new era of targeted therapeutics applicable in principle to other transporter-related diseases, including those relevant to placental biology (7).

While these isoforms are recognized for protecting cancer stem cells from chemotherapy, they also fundamentally dictate systemic drug pharmacokinetics by governing the absorption, distribution, and elimination of various agents through competitive efflux at critical physiological barriers (43). Furthermore, genetic polymorphisms within these transporters can modulate their expression or functional activity, contributing to significant inter-individual variability in drug disposition and potentially influencing therapeutic efficacy or the incidence of adverse drug reactions (44–46). Consequently, the characterization of these genetic variants remains a clinical priority for predicting patient response to drugs that are substrates for specific ABC proteins, such as ABCG2-mediated renal clearance of uric acid or toxic metabolites (32). Beyond their physiological role, the aberrant upregulation of these proteins in cancer stem cells serves as a primary mechanism of multidrug resistance by facilitating the rapid extrusion of structurally diverse chemotherapeutic agents (47, 48). These efflux pumps not only facilitate intrinsic resistance but also promote cross-resistance to varied classes of therapeutics, including anthracyclines and tyrosine kinase inhibitors (49). Furthermore, post-transcriptional regulation, such as the miR-212-mediated regulation of ABCG2, add another layer of complexity to these resistance phenotypes by dynamically altering transporter expression levels in response to prolonged therapeutic exposure (50). In addition, these transporters are subject to post-translational regulation, where protein degradation pathways directly modulate surface expression and efflux capacity. For instance, the ubiquitin-proteasome system governs the stability of proteins like ABCB1, where enhanced ubiquitination leads to accelerated proteasomal degradation and a subsequent reduction in functional efflux (51). Similarly, the turnover of transporters such as ABCG2 is differentially managed, with wild-type protein primarily subject to lysosomal sorting, while misfolded or mutant variants are frequently diverted to the proteasome for degradation (52, 53). This post-translational control, which is modulated by factors including cellular stress and metabolite levels, represents a critical, rapid-acting mechanism for fine-tuning transporter abundance at the plasma membrane, operating independently of the slower timescales of transcriptional regulation (51). In short, the precise calibration of these transporters at the plasma membrane serves as a dynamic metabolic checkpoint that dictates both the barrier function of healthy tissues and the chemosensitivity of malignant cells.

## 3 Role of ABC transporters in the placenta

The human placenta features a hemochorial architecture where villous trophoblast trees composed of an outer multinucleated syncytiotrophoblast layer and an inner cytotrophoblast layer are bathed directly in maternal blood to separate maternal and fetal circulations across a specialized trophoblast-based placental barrier (1, 2). Transplacental passage occurs exclusively across the STB for most small molecules, and the presence of ABC efflux transporters on the apical brush-border membrane of the STB (facing maternal blood) is the primary molecular mechanism by which the placenta limits fetal drug and toxin exposure (54).

**Figure 2:**
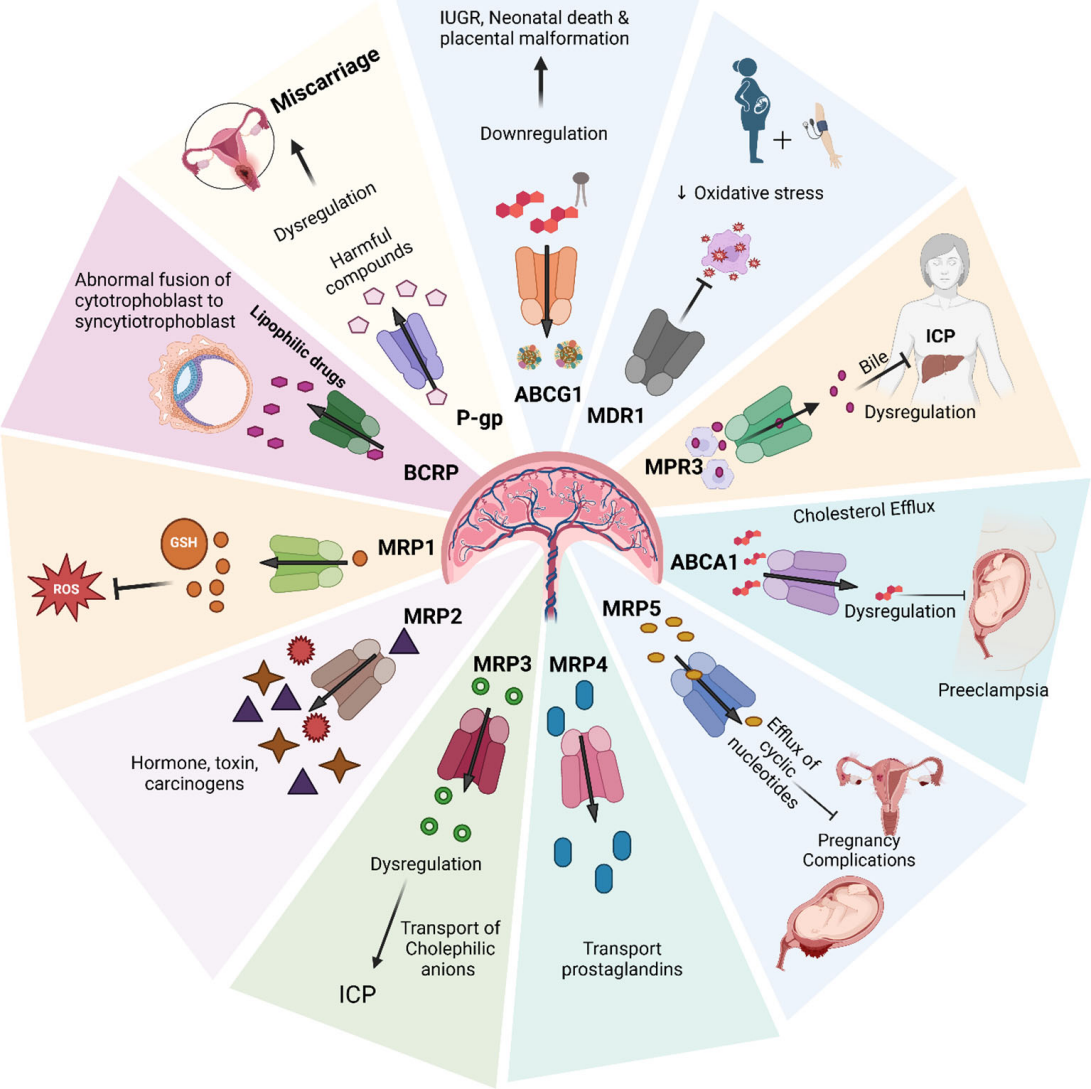
Role of different ABC transporters in placenta and their function in maintaining pregnancy. BCRP is expressed on fetal endothelium, CTB, STB which are present on the apical micro-villous surface that transports different nutrients and restricts xenobiotics and harmful drugs from maternal blood across the apical membrane to the fetal circulatory system. BCRP decreases the xenobiotics concentrations entering from maternal blood into fetal blood. The substrates for BCRP include porphyrins, riboflavin, estrogens, mitoxantrone, bile acids, and glyburide. MRP1 is present in the basolateral membrane and involved in the reduction of MeHg-induced oxidative stress produced by (ROS) through exporting ROS from inside of cells in the form of glutathione conjugates. MRP2 transporters export hormones, toxins, and carcinogens. MRP3 present on the apical membrane of syncytiotrophoblasts, exports cholephillic anions. Any dysregulation in its expression leads to the development of ICP. MRP4 transport prostaglandins. MDR3’s function is to transport phosphatidylcholines and it is also involved in the transport of many substrates including ivermectine, vinblastine, paclitaxel, and digoxin. MRP5 is present in the basal membrane of syncytiotrophoblasts involved in the transport of cyclic nucleotides. ABCA1 is present on the apical membrane of syncytiotrophoblasts and involved in the transport of cholesterol and phosphatidylcholine lipids. Dysregulation of its expression leads to preeclampsia in pregnant women. MPR3 is involved in the excretion of biliary phospholipids from hepatocytes into bile and lack of its excretion leads to the progression of ICP. MDR1 causes a reduction of oxidative stress by the export of ROS from cells while a decrease in its expression leads to preeclampsia. ABCG1 exports cholesterol and PC family lipids and abnormality in these functions is subsequent in IUGR, neonatal death & placental malformation. P-gp exports xenobiotics from fetal cells and prevents cells from exposure to these harmful compounds. Any dysregulation leads to spontaneous miscarriage or abortion.

Besides drug efflux, the physiological functions of placental ABC proteins are critical as well (**Figure 2**). ABCA1 mediates cholesterol and phosphatidylcholine secretion into ApoA-I, necessary for cholesterol regulation in the placenta and lipid transfer to the developing fetus. While ABCG1 and ABCG4 control the levels of cellular cholesterol and lipid composition in membranes, the MRP3 (ABCC3) transports bile acids from syncytiotrophoblasts toward maternal blood. The BCRP/ABCG2 is involved in resistance against plant-derived estrogens, toxins, and environmental contaminants like bisphenol-A (9, 55, 56). Additionally, the endocrine function of the placenta estrogen is coupled to ABC transporter-mediated steroid hormone efflux, particularly by P-gp and BCRP (8). Lye et al. (25) reported a potentially important novel function for BCRP in early placentation. Extravillous trophoblast (EVT) invade the maternal decidua and remodel uterine spiral arteries in a process essential for establishing low-resistance placental perfusion, and their dysregulation underlies PE and (IUGR). Using siRNA-mediated ABCG2 knockdown in the HTR8/SVneo EVT-like cell line and in first trimester human placental explants (6–7 weeks gestation). Lye et al. further demonstrated that BCRP inhibits EVT cell migration without affecting proliferation, and confirmed BCRP protein expression in EVTs by immunohistochemistry (25). The same study showed that bacterial (LPS) and viral (single-stranded RNA) stimuli markedly downregulated ABCG2/BCRP in EVTs while concurrently increasing EVT migration potential. This supports the notion that infection-driven BCRP downregulation could disrupt normal trophoblast migration dynamics in ways that may be detrimental to early placentation (25). Importantly, the authors explicitly acknowledged that information about BCRP function in EVTs is ‘limited’. Therefore, independent replication in additional model systems or *in vivo* infection contexts has not yet been reported. Furthermore, the mechanism’s relevance beyond early first trimester remains to be determined recognize.

The expression of placental ABC transporters is heterogeneous across trophoblast subtypes and shifts substantially with gestational age. At the level of membrane polarity within the (STB), ABCB1, ABCG2, ABCC2, ABCC3, and ABCA1 localize predominantly to the apical brush-border membrane facing maternal blood, constituting the primary efflux barrier; in contrast, ABCC1 and ABCB4 occupy the basolateral membrane interfacing with the villous stroma and fetal capillaries (57, 58). At the level of trophoblast subtype specificity, ABCG2/BCRP is uniquely expressed across STB, CTB, and EVT and perform distinct roles in xenobiotic and drug efflux at the STB apical surface, modulation of CTB-to-STB syncytialization, and restraint of EVT invasion (59). ABCB1/P-gp is also present in CTB and EVT, where functional studies demonstrate a role in supporting EVT invasion and trophoblast differentiation (59). This an activity independent of its STB xenobiotic efflux function and specifically reduced in the PE placenta. ABCA1 is expressed in both STB (apical membrane) and CTB, mediating reverse cholesterol transport to ApoA-I acceptors, while ABCG1 is present in STB cholesterol-rich membrane domains and in CTB undergoing syncytialization (60). At the level of gestational dynamics, ABCB1 protein is highest in the first trimester and declines progressively across gestation, consistent with heightened fetal vulnerability during organogenesis (61, 62). Conversely, ABCG2 protein levels rise toward term despite stable mRNA expression, a pattern indicative of post-transcriptional regulation, such as miRNA-mediated post-transcriptional regulation (63). ABCA1 and ABCC3 mRNA both increase with advancing gestation in healthy placentas (64), placing disease-associated reductions (e.g., in PE and IUGR) within the context of a normally ascending expression trajectory that PE disrupts rather than simply reverses.

To evaluate the role of ABC transporters in human placenta and associated pregnancy complications, we applied three criteria on 48 ABC transporter genes. First, we required experimental evidence of protein-level expression in at least one human placental trophoblast compartment including the STB, CTB, EVT. Second, we required documented functional role in materno-fetal exchange, trophoblast biology, or placental physiology demonstrated in primary human trophoblast or *in vivo* studies. Third, we required reported association with at least one pregnancy complication based on human cohort or animal model studies. The remaining 40 members of the ABC superfamily either lack documented trophoblast expression, are confined to non-trophoblast placental cells such as endothelial cells, Hofbauer macrophages, or decidual cells, or have not been studied in any pregnancy-related context. A summary of the findings is presented in **Table 2**.

**Table 2:** Pathophysiology of ABC transporters in human placenta and associated pregnancy complications.

ABC transporter	Localization in placenta	Expression level	Physiological function	Pregnancy-related dysregulation	Key references
P-gp/ABCB1	Apical membrane of STB; fetal capillary endothelium*; CTB; EVT*	High in early gestation; generally decreases with advancing gestation	Efflux of xenobiotics, steroid hormones, and cytotoxic compounds into maternal circulation; protects fetal compartment	↑ in preterm birth; ↓ leads to spontaneous miscarriage; altered by LPS/infection; reduced in severe PE	(1, 8, 65, 66)
BCRP/ABCG2	Apical brush-border membrane of STB; fetal endothelium; EVTs	Highly expressed/abundant in placenta; gestation- and cell-type-dependent	Efflux of lipophilic drugs, bile acids, porphyrins, riboflavin, glyburide; regulation of EVT migration and cytotrophoblast fusion	↓ in PE & IUGR; ↓ under hypoxia (HIF-1α); suppressed by TNF-α and IL-1β; ↑ in early miscarriage	(1, 25, 67, 68)
MRP1/ABCC1	Basolateral membrane of STB	Low–moderate	Export of GSH-conjugated toxic metals (e.g. MeHg), reduction of oxidative stress; organic anion efflux	↓ in preeclampsia (oxidative stress amplified); ↓ in malaria in pregnancy models; role in protection against MeHg fetotoxicity	(56, 69, 70)
MRP2/ABCC2	Apical membrane of STB	Moderate	Export of bilirubin conjugates, hormones, organic anions, toxins, and carcinogens; bile-acid-related	↑ mRNA in ICP patients; ↓ in rat preeclampsia model; reversed by Nrf2 activation	(11, 54, 71)
MRP3/ABCC3	Apical membrane of STB	High in placenta	Transport of organic cholephilic anions; glyburide efflux from fetal to maternal compartment	Dysregulation contributes to cholestatic liver disease and ICP; altered in gestational diabetes	(24, 72, 73)
MDR3/ABCB4	Basolateral membrane of STB; hepatic canalicular membrane	Low in placenta; high in liver	Phosphatidylcholine flippase; efflux of certain drug substrates into fetal capillaries	ABCB4 variants are linked to ICP and PFIC3; placental drug-transfer relevance remains uncertain	(4, 22, 23, 74)
ABCA1	Apical membrane of STB; cytotrophoblasts; endothelial cells	Gestational age dependent; higher in preterm placenta	Reverse cholesterol transport; export of cholesterol and phosphatidylcholine to apolipoprotein acceptors; critical for placental and fetal lipid homeostasis	↓ mRNA linked to higher risk of PE; ABCA1 deficiency causes placental malformations, retarded embryo growth, and miscarriage in rodents	(9, 21, 75, 76)
ABCG1	Apical membrane of STB; cholesterol-rich membrane domains	Moderate	Cholesterol and PC family lipid export; lipid homeostasis; modulates TCR signaling in T lymphocytes	↓ in PE and GDM; ABCG1 deficiency causes IUGR, neonatal death, and placental malformation; ↓ in early miscarriage linked to reduced cholesterol export to fetus	(9, 10, 77, 78)

Abbreviations: BCRP, breast cancer resistance protein; CTB, cytotrophoblast; EVT, extravillous trophoblast; GDM, gestational diabetes mellitus; GSH, glutathione; HIF-1α, hypoxia-inducible factor 1-alpha; ICP, intrahepatic cholestasis of pregnancy; IL-1β, interleukin-1 beta; IUGR, intrauterine growth restriction; LPS, lipopolysaccharide; MDR3, multidrug resistance protein 3; MeHg, methylmercury; mRNA, messenger RNA; MRP1/MRP2/MRP3, multidrug resistance-associated protein 1/2/3; Nrf2, nuclear factor erythroid 2-related factor 2; PFIC3, progressive familial intrahepatic cholestasis type 3; P-gp, P-glycoprotein; TCR, T-cell receptor; TNF-α, tumor necrosis factor-alpha.

↟ = increased/upregulated; ↡ = decreased/downregulated.

## 4 ABC transporters in placental and systemic immune responses

In addition to xenobiotic efflux, the physiology of ABC transporters in immunological homeostasis are pertinent to pregnancy, where immune tolerance toward the semiallogenic fetus needs to be regulated carefully. ABCG1 regulates cholesterol in the plasma membrane of T lymphocytes. When ABCG1 is absent, cholesterol accumulates in these cells, which leads to increased signaling through the T cell receptor. This heightened signaling manifests as increased phosphorylation of ZAP-70 and ERK1/2. The resulting dysregulation causes developmental defects in thymocytes and promotes autoimmunity signaling (79, 80). Increased T cell signaling during pregnancy, such as aberrant Th17 polarization at the interface between the mother and fetus, has been associated with recurrent miscarriage and preeclampsia which indicates the involvement of lipid homeostasis in immune cells mediated by ABCG1 in maintaining a tolerant uterine milieu (80, 81). Furthermore, ABCB1/P-gp shows significant accumulation in the tumor microenvironment where it controls the cytokine production by immune cells, especially affecting CD8+ T cell memory and differentiation (82). Regarding the effect of infectious diseases on pregnancy, viruses (such as the Zika virus, CMV, and SARS-CoV-2) tend to inhibit BCRP and P-gp in the placenta and predispose the fetus to exposure to maternal drugs and inflammation (74, 83). It has been established that the downregulation of BCRP through TNF-α and IL-1β, which are highly abundant during PE and GDM, occurs in primary term trophoblasts (1, 8).

## 5 Mutations in ABC transporter genes and pregnancy complications

### 5.1 Intrahepatic cholestasis of pregnancy

ICP, otherwise known as obstetric cholestasis, is the most common pregnancy-specific liver disorder, characterized by the onset of severe itching (primarily at night), elevated maternal serum bile acids, with severity thresholds varying by guideline, and high levels of liver enzymes. The condition is reversible post-delivery, although there are serious risks associated with poor outcomes for the fetus such as spontaneous preterm birth, meconium-stained amniotic fluid, fetal distress, and stillbirth (71, 84, 85). Its prevalence is relatively higher in Scandinavia, South America, particularly Chile and Bolivia, and South Asia, with pronounced seasonal differences and recurrence rates of 40–60% in future pregnancies (86).

Molecular mechanisms involved in the pathogenesis of ICP are based on the malfunction of hepatobiliary and placental bile acid (BA) transporters. During normal pregnancy, there is a gradient of bile acid concentration between fetus and mother; in healthy conditions, BAs are transferred from fetus to mother through placental bile acid transport systems, including ABC and SLC transporters, which transfers BAs from fetus to mother. However, in ICP, the reverse gradient of BA concentration occurs, leading to the accumulation of potentially harmful agents inside the fetus. Placental ABC transporters (mainly MRP3 (ABCC3) and BCRP) have shown upregulation as a defensive reaction, but not in severe cases of ICP (87).

Sequencing-based genetic studies have identified rare and common variants in ABCB4 (MDR3), ABCB11 (BSEP/bile salt export pump), and ABCC2 (MRP2) in ICP patients (71). Numerous ABCB4 variants have been reported, and a subset is associated with ICP susceptibility, and mutations in ABCB4 on chromosome 7q21 are one of the most recognized causes of genetic susceptibility to familial ICP type 3 (22, 23). Disruption of ABCB4-encoded phosphatidylcholine (PC) flippase function leads to an inhibition of PC secretion in the biliary lumen, impairing bile salt-induced micelles formation and causing cholestatic damage to biliary epithelial cells and hepatocytes (88, 89). ABCB11 malfunction results in impaired active bile salt secretion into bile canaliculi, leading to their intracellular accumulation with toxic effects. Additionally, a case-control study demonstrated that ICP placentas have increased ABCC2 mRNA expression, indicating upregulation of MRP2-dependent bilirubin conjugates secretion (71). Recent genomic investigations highlight that pathogenic heterozygous mutations in both ABCB4 and ABCB11 are identifiable in up to 20-25% of severe, early-onset ICP cases (90). Furthermore, the functional impairment of ABCC2 has been linked to the altered excretion of conjugated estradiol-17β-D-glucuronide, which can trans-inhibit bile salt export pumps and exacerbate cholestatic conditions (91). Beyond rare monogenic variations, large scale genome-wide association studies have identified multiple common loci, including ABCB11 and ABCG8, that contribute to the polygenic architecture and ICP susceptibility and bile acid/lipid metabolic regulation (90, 92). Overall, these findings underscore the broader aspects of interaction of metabolic trait-associated loci with impaired BA homeostasis, as excessive synthesis often correlates with suppressed FXR signaling pathways.

### 5.2 Preterm birth

Preterm birth, defined as delivery before 37 weeks of gestation, remains the leading cause of neonatal death globally. Preterm birth complications are responsible for approximately 900,000 under-five deaths annually. Survivors face significant long-term morbidity including cognitive developmental delays, respiratory disease, and cardiovascular pathology among (14, 65). The causes of PTB are diverse, ranging from infections and inflammation during pregnancy, placental insufficiency, decidual bleeding, uterine overdistention, iatrogenic preterm birth, to unknown etiologies in many situations (93).

In a study group, Scott et al. (65) showed that PTB is linked to higher placental mRNA and protein expression levels of ABCB1 (P-gp) and, to a lesser extent, ABCG2 (BCRP), independent of maternal body mass index before conception. This finding is mechanistically plausible as the elevated P-gp and BCRP in preterm placentas may reflect a compensatory upregulation to protect against the increased endogenous inflammatory substrates (including prostaglandins, whose transport by MRP4/ABCC4 also plays an important role) and exogenous xenobiotics present in the inflammatory milieu associated with preterm parturition (56, 65). A specific infectious trigger was demonstrated by Fontes et al. (56) in a murine model of *Plasmodium berghei* malaria-induced PTB, where placental ABC transporters (e.g., including ABCB1 and ABCG2) were significantly dysregulated. This study provides a direct mechanistic evidence linking placental ABC transporter dysfunction to infection-driven preterm labor (56).

Justesen et al. (61) further reported that ABCB1 expression is elevated in first trimester placentas of overweight (BMI 25-29.9 kg/m^2^) and obese (BMI ≥ 30 kg/m^2^) pregnant women, suggesting that maternal metabolic status modulates the placental ABC transporter landscape early in gestation, potentially predisposing to adverse outcomes including PTB (61). BCRP can limit fetal exposure to selected drugs used or encountered during pregnancy, including glyburide; other transporters such as P-gp/ABCB1 are more relevant for substrates such as digoxin. BCRP regulate cytotrophoblast–syncytiotrophoblast fusion. Disruption of BCRP function in the preterm setting carries important pharmacological implications for antenatal drug administration strategies (67, 94). Moreover, polymorphisms in the ABCC2 gene have been correlated with altered detoxification of placental xenobiotics and endogenous metabolites, which may further modulate the timing of parturition (95). Additionally, recent data suggest that suboptimal efflux of prostaglandins and other pro-contractile mediators across the placental barrier, mediated by ABC transporter substrates, may lower the threshold for labor induction (96). Beyond these efflux mechanisms, the placental barrier function relies significantly on the expression of ABCB1 and ABCC1, which maintain fetal homeostasis by restricting the passage of maternal glucocorticoids and toxins (97). Reduced expression or inhibition of these transporters compromises the fetal-maternal physiological barrier, thereby increasing the vulnerability of the placental unit to systemic inflammation and premature cervical ripening. In short, dysfunction in these transport systems contributes to a proinflammatory environment at the maternal-fetal interface that potentially triggers the cascade of preterm birth.

### 5.3 Preeclampsia

Preeclampsia is a leading cause of maternal and perinatal morbidity and mortality worldwide, complicating 3–8% of pregnancies and contributing substantially to global maternal and perinatal deaths (12, 13). The underlying pathophysiology centers on defective early placentation, specifically impaired EVT invasion and failure to remodel the uterine spiral arteries into high-flow, low-resistance vessels, leading to chronic placental oxidative stress, endothelial dysfunction, and multi-organ dysfunction in the mother (13, 98).

Multiple studies have documented significant dysregulation of cholesterol-transporting ABC transporters in PE placenta. Sethuraman et al. (9) found that ABCA1, ABCG1, ABCG5, and ABCG8 mRNA expression is altered during syncytialization in preeclamptic placentas, implicating dysregulated reverse cholesterol transport in placental pathology (9). Specifically, Wolski et al. (21) found that downregulation of ABCA1 occurs in the placentas of patients with late-onset PE (21), similar to previous findings where Liu et al. (75) demonstrated that low serum ABCA1 levels predicted PE (75). These apparently contradictory findings can be reconciled by considering cell type-specific expression dynamics and PE phenotypic heterogeneity. At the level of the mature STB, the terminally differentiated cholesterol-exporting syncytium, ABCA1 protein is specifically reduced in PE as demonstrated by Baumann et al. (60), who showed that PE selectively affects ABCA1 levels in syncytiotrophoblasts (60). In contrast, Sethuraman et al. (9) showed that static whole-tissue ABCA1 protein and mRNA expression remain unaltered in term-PE placentas overall, while the dynamic induction of ABCA1 and ABCG1 mRNA during CTB-to-STB syncytialization is significantly amplified in term-PE-derived CTBs (after 96h of fusion) compared with healthy term controls (9). This finding suggests that ABCA1 dysregulation varies by PE phenotype and trophoblast differentiation stage. Furthermore, ABCA1 is reduced in mature STB but becomes aberrantly elevated during the cytotrophoblast-to-syncytiotrophoblast transition. Hypoxia compounds this dysregulation (99). Early-onset PE involves greater placental hypoxia due to failed spiral artery remodeling, which activates HIF-1α and further suppresses ABCA1 in STB (100). CTBs appear to mount a compensatory upregulation response in an attempt to maintain adequate cholesterol efflux capacity (101). Finally, most existing studies measured ABCA1 in unseparated placental homogenates and recruited heterogeneous PE cohorts (early- versus late-onset, mild versus severe, varying gestational ages), therefore, there are methodological differences in the current literature that collectively account for the discordant literature. Resolving these contradictions will require studies employing laser-capture microdissection or single-cell RNA sequencing with rigorous stratification by PE subtype and trophoblast compartment.

The importance of nuclear factor NRF2 as an upstream regulatory element connecting the oxidative stress with the downregulation of ABC transporters in PE cannot be overstated. Specifically, Yu et al. (11) showed that in a PE rat model, BCRP, MRP1, MRP2, and P-gp were all downregulated in preeclamptic rat placentas, where NRF2 upregulation restored ABC transporter expression alongside antioxidant gene activation and relieved hypertension in the same animal model (11). In a study using primary human trophoblasts and human PE placental tissue, Fuenzalida et al. (98) investigated cholesterol transporter responses to hypoxia and found that HIF-1α and NRF2 produce complex, context-dependent changes in cholesterol-transporting ABC transporters. In primary human trophoblasts, ABCA1 mRNA was paradoxically increased under hypoxia (a potential compensatory response), whereas ABCA1 protein was reduced in human PE placental tissue, suggesting disrupted cholesterol homeostasis whose directionality differs between cell state and disease compartment. These findings caution against interpreting placental hypoxia as uniformly suppressing cholesterol transporters’ ability to efflux lipids in preeclampsia (98). Extrapolating from the rodent model and *in vitro* data above, MDR1/P-gp downregulation in the PE placenta may impair clearance of oxidatively-modified lipid substrates from syncytiotrophoblasts, and amplify oxidative damage, although direct evidence for this mechanism in human PE clinical samples is currently limited. Meanwhile, downregulation of ABCA1 and ABCG1 further increases the cholesterol imbalance in PE patients (98, 102). Furthermore, the suppression of the LAT1-NRF2 axis has been identified as a critical upstream event, linking reduced transporter activity to the characteristic anti-angiogenic sFlt-1/PlGF imbalance observed in clinical PE (103). Consequently, the therapeutic restoration of NRF2 activity may offer a dual-protective strategy by simultaneously mitigating oxidative injury and correcting the anti-angiogenic signaling profile (103). Beyond this regulatory crosstalk, NRF2 is known to promote cellular efflux by directly upregulating the expression of various ABC transporters including MDR1 and BCRP (104). In short, the dysregulated NRF2-ABC transporter axis serves as a critical node where chronic oxidative stress and placental hypoxia converge to exacerbate the metabolic and anti-angiogenic features of PE (105, 106).

### 5.4 Early miscarriage

Spontaneous abortion prior to 20 weeks of gestation has been estimated to affect between 10–20% of all recognized pregnancies, being one of the commonest complications of human reproduction (16). Out of recognized clinical pregnancies, around 80% of the losses occur within the first trimester period. Around 50-60% of first trimester losses have been attributed to chromosomal anomalies. However, other factors like endometrial, endocrine, immune, thrombophilic, and environmental factors play their role in remaining abortions (107, 108).

According to Lanjewar et al. (66), another landmark study on first trimester spontaneous abortion revealed that loss of expression of P-glycoprotein in placenta could be one possible convergent pathophysiological mechanism behind first trimester spontaneous abortions. The researchers found through immunohistology that the loss of first trimester miscarried placentas had significantly low P-gp levels compared with normal first trimester placentas (66). Mechanistically, high levels of P-gp proteins are needed in early placental trophoblasts to protect the fetus from exposure to various potentially teratogenic molecules in the form of hormones, xenobiotics, and cytokines. Loss of these molecules leads to decreased protection against any potential teratogens and genotoxins (66).

Shahnawaz et al. (78) further built on this framework by showing that dysfunctional autophagy in early miscarriages led to simultaneous upregulation of ABCG2 and downregulation of ABCG1 in placentas (78). Autophagy dysfunction increases accumulation of oxidized lipids and ROS generation, which causes increased levels of ABCG2 as a protective compensatory response. Downregulation of ABCG1 results in decreased capacity of placenta to move out cholesterol to the fetus and thus reduces fetal cholesterol. Moreover, a rodent study shows that lack of Abca1 protein leads to placental developmental problems as well as retarded fetal growth and subsequent miscarriage (76). Such molecular disruptions in efflux transporters are often observed alongside broader, complex etiologies, including anatomical anomalies and maternal immune responses, that collectively contribute to the high clinical heterogeneity of recurrent pregnancy loss (109). Furthermore, advancements in diagnostic techniques have underscored those chromosomal aberrations, such as trisomies or monosomy X, often coexist with these transporter-related disruptions, complicating the clinical assessment of idiopathic miscarriages (16, 110). Overall, these transporter-linked deficiencies highlight a critical intersection where impaired placental detoxification and altered nutrient transport susceptibility significantly elevate the risk of pregnancy termination beyond purely genetic factors.

### 5.5 Gestational diabetes mellitus

GDM is described as a form of glucose intolerance diagnosed for the first time during pregnancy. It has been estimated that GDM affects about 14% of all pregnancies globally and approximately 5–9% of pregnancies annually in the United States, with its incidence rising concurrently with the increase in the incidence of obesity worldwide (15, 111). In terms of pathophysiology, GDM arises due to a combination of insulin resistance and decreased insulin production capacity by the maternal pancreatic beta cells. Fetal complications include macrosomia, neonatal hypoglycemia, and shoulder dystocia, while the maternal and child risks associated with GDM are obesity, type 2 DM, and cardiovascular disease (112, 113).

ABCG1 appears to be the ABC transporter most directly involved in GDM pathogenesis. In a human genetic association study, Liu et al. (77) identified ABCG1 variants that significantly increase GDM susceptibility. They noted that ABCG1 deficiency causes massive lipid deposition in different organs and tissues and plays a crucial role in regulating high-density lipoprotein metabolism and cholesterol efflux. Epigenetic control of ABCG1 activity modulates the interconnection between glucose metabolism and cholesterol metabolism, suggesting that ABCG1 is an epigenetic regulator of both type 2 diabetes and GDM (77).

In terms of drug therapy for GDM, the oral anti-diabetes medication glyburide (also known as glibenclamide) is frequently employed as an insulin replacement. Importantly, glyburide is transported by BCRP/ABCG2 and MRP3/ABCC3, which are expressed by the STB. Interestingly, *in vitro* transport assays using BCRP- and MRP3-overexpressing cell lines demonstrated that both transporters actively efflux glyburide, identifying them as candidate regulators of transplacental glyburide passage (24). Subsequent *ex vivo* work using human placental brush-border membrane vesicles confirmed that BCRP mediates glyburide transfer (114), and *in vivo* data from ABCG2-knockout pregnant mice, in which fetal glyburide concentrations are approximately 2-fold higher than in wild-type controls, further corroborate the physiological relevance of placental BCRP-mediated fetal protection from glyburide (24). Therefore, if GDM-associated reductions in placental BCRP or MRP3 expression occur, a mechanistic prediction is that fetal glyburide exposure would increase. This concern is supported by the ABCG2-knockout mouse data above (8, 24), but not yet confirmed in GDM patients receiving glyburide. Furthermore, clinical reliance on pharmacological interventions for GDM remains complex, as glyburide has been associated with higher rates of neonatal hypoglycemia and macrosomia compared to insulin or metformin (115). Furthermore, research indicates that altered methylation levels of the ABCA1 transporter gene in the placenta are associated with maternal glucose levels and HDL cholesterol, suggesting that epigenetic dysregulation of cholesterol efflux transporters contributes to the metabolic landscape of GDM (116). Additionally, emerging evidence highlights that ABCG1 may represent an important node in these metabolic pathways, linking cholesterol/phospholipid transport with broader glucose and lipid dysregulation (117). Overall, further research into the molecular mechanisms of GDM must integrate these genetic findings with clinical strategies, such as assessing placental miRNA profiles, e.g., miR-122-5p, which may modulate metabolic pathways related to glucose and lipid homeostasis. These molecular insights emphasize the potential for developing precision-based management strategies that account for both maternal genetic variability and placental transport capacity.

### 5.6 Intrauterine growth restriction

IUGR, characterized by the inability of the fetus to reach its genetically predetermined size due to a pathological limitation leading to low birth weight under the 10th percentile mark, occurs in about 5-10% of all pregnancies across the world and remains a significant risk factor for both perinatal mortality and morbidity as well as development of non-communicable diseases in adults (118, 119). Most often, the reason behind IUGR is placental insufficiency and poor uteroplacental perfusion causing lack of oxygen and nutrients supply for the fetus.

Currently, the strongest evidence connecting ABC transporters with IUGR concerns the role played by ABCA1. According to Winterhager & Gellhaus (76), the lack of Abca1 gene expression in mice leads to abnormal placental development marked by malformations of the labyrinth vascularization and layers of trophoblasts with subsequent occurrence of IUGR and high levels of embryonic lethality (76). The exact mechanism includes ABCA1-mediated cholesterol transfer from trophoblast cells into the blood of the fetus which allows regulating the function of the cholesterol-sensitive Hedgehog and Wnt signaling pathways involved in the process of placental morphogenesis. In addition, suppression of BCRP in IUGR cases associated with chronic hypoxia and activation of HIF-1α increases vulnerability of the placenta to apoptotic stimuli (1, 98). Furthermore, the placental expression of ABCB1 and ABCC1 facilitates the modulation of the fetal glucocorticoid barrier, although the main placental glucocorticoid barrier is mediated by 11β-HSD2 during critical growth windows (97). Consequently, alterations in the expression or function of these specific ABC transporters may further compromise placental efficiency and directly contribute to the restricted nutrient and oxygen exchange that defines the pathogenesis of IUGR. Given that suboptimal placentation is a primary driver of IUGR (120), the disruption of these efflux pumps potentially exacerbates the physiological consequences of chronic hypoxia and nutrient insufficiency (121). Overall, these genetic variations and functional impairments in placental transporter systems represent a pivotal, often overlooked, molecular etiology that links maternal metabolic status to long-term fetal growth trajectories and postnatal health outcomes. Future research should focus on how these transporter-mediated deficits interact with periconceptional environmental cues to fix heritable chromatin changes that dictate long-term metabolic health.

We have summarized associations between dysregulation of ABC transporters and major pregnancy-related disorders in **Table 3**.

**Table 3:** Summary of pregnancy complications, associated ABC transporter dysregulation, prevalence, maternal symptoms, and fetal effects.

Condition	ABC transporter dysregulation	Global prevalence	Maternal symptoms	Fetal/neonatal effects	References
ICP	ABCB4/MDR3 and ABCB11/BSEP variants or reduced hepatobiliary function; ↑ placental ABCC2/MRP2 reported	Variable globally; higher rates reported in Scandinavia, South America, particularly Chile/Bolivia, and South Asia	Pruritus (especially nocturnal), insomnia, elevated serum bile acids, elevated liver enzymes, jaundice (rare)	Spontaneous preterm delivery, fetal distress, meconium staining, respiratory distress, poor Apgar scores, increased stillbirth risk	(22, 71, 84, 87)
Preterm Birth (PTB)	↑ ABCB1 (P-gp) ↑ ABCG2 (BCRP)	~10% of births worldwide; 13.4 million preterm births in 2020; ~900,000 under-five deaths from preterm complications in 2019	Uterine contractions before 37 weeks, cervical dilation, membrane rupture	Neurodevelopmental morbidity, sensorineural deficits, respiratory distress syndrome, IVH, NEC, increased risk of adult cardiovascular disease	(1, 14, 56, 65)
PE (PE)	↓ ABCA1, ↓ ABCB1 ↓ ABCG1, ↓ ABCG2 ↓ ABCC1, ↓ ABCC2	~3–8% of pregnancies worldwide; major contributor to maternal and perinatal morbidity/mortality	New-onset hypertension (≥140/90), proteinuria, edema, headache, visual changes, multi-organ dysfunction	IUGR, oligohydramnios, placental abruption, premature delivery, fetal distress, increased stillbirth risk; long-term cardiovascular risk for offspring	(9, 11, 13, 21, 98)
Early Miscarriage	↓ ABCA1, ↓ ABCB1 ↓ ABCG1, ↑ ABCG2	~10–20% of clinically recognized pregnancies; higher when very early/unrecognized losses are included	Vaginal bleeding, pelvic pain, passage of fetal tissue; psychological distress, depression, PTSD	Pregnancy loss before 20 weeks; fetal malformation or aneuploidy; no cardiac activity on ultrasound	(16, 66, 78, 107)
GDM	↓ ABCG1, ↓ ABCG2 ↓ ABCC3 (MRP3)	~14% globally, varying by population and diagnostic criteria; ~5–9% of U.S. pregnancies annually	Hyperglycemia, polyuria, polydipsia, fatigue; often asymptomatic (detected by OGTT)	Macrosomia, neonatal hypoglycemia, respiratory distress, risk of childhood obesity, insulin resistance, and type 2 DM in offspring	(15, 24, 77, 113)
IUGR (IUGR)	↓ ABCA1	5–10% of pregnancies;	Reduced uterine fundal height, oligohydramnios, decreased fetal movements, abnormal Doppler flows	Low birth weight (<10th percentile), perinatal asphyxia; long-term: metabolic syndrome, obesity, type 2 DM, hypertension, cardiovascular disease	(76, 118, 119, 122)
Malaria in pregnancy (MiP)	↓ P-gp (ABCB1) ↓ BCRP (ABCG2) ↓ MRP1/2/3	~121.9 million pregnancies occurred in malaria transmission areas in 2020; burden highest in endemic regions, especially sub-Saharan Africa	Fever, severe anemia, hypoglycemia, placental sequestration of P. falciparum, risk of cerebral malaria and pulmonary edema	Low birth weight (<2500 g), IUGR, preterm delivery, neonatal mortality; impaired childhood cognitive development	(56, 123–125)

Abbreviations: BCRP, breast cancer resistance protein; BSEP, bile salt export pump; DM, diabetes mellitus; ICP, intrahepatic cholestasis of pregnancy; IUGR, intrauterine growth restriction; IVH, intraventricular hemorrhage; MDR3, multidrug resistance protein 3; MiP, malaria in pregnancy; MRP1/MRP2/MRP3, multidrug resistance-associated protein 1/2/3; NEC, necrotizing enterocolitis; OGTT, oral glucose tolerance test; PE, preeclampsia; P. falciparum, Plasmodium falciparum; PTB, preterm birth; PTSD, post-traumatic stress disorder.

↟ = increased/upregulated; ↡ = decreased/downregulated; ≤ = greater than or equal to; < = less than.

## 6 Emerging technologies in placental ABC transporters biology

The past years have seen important progress made in the field of structural, translational, and clinical knowledge of placental ABC transporters. One such breakthrough study is a review by Alam & Locher (4) on the structural basis of ABC subfamily transporters’ substrate specificity, ATP binding and catalysis, and the mechanisms underlying mutations linked to pathogenic conditions (4). These structural studies are highly relevant to the placenta field, specifically, knowing the atomic structure of how phosphatidylcholine flips in ABCB4 (MDR3) would be a useful tool in developing drug candidates capable of stabilizing mutant ABCB4 responsible for ICP (7).

With regards to translation, Moore et al. (7) published a comprehensive review on ABC transporter-related disorders and recent developments in the pharmacotherapy of these conditions, including the success story of the CFTR modulator drugs as a paradigm (7). Developing small molecule drugs to enhance the expression and function of ABC transporters in preeclamptic, GDM, and/or ICP pregnancies is an important new direction with clear clinical relevance. Concurrently, Kotta-Loizou et al. (2024) offered an in-depth view into the latest methodology in terms of placenta-on-a-chip technology, physiological-based pharmacokinetics of drugs between maternal and fetal circulation, and trophoblast organoids that are becoming an integral part of such transplacental drug transport prediction tools (126). Beyond the structural and pharmacological advances summarized in **Table 4**, the emergence of single-cell and spatial transcriptomics is beginning to provide the cell type resolution that bulk placental studies cannot supply. Single-cell RNA sequencing (scRNA-seq) of human first trimester placentas has revealed that ABCB1, ABCG2, and cholesterol-transporting ABC transporters are expressed heterogeneously across (CTB), (STB), and (EVT) subpopulations, such that many disease-associated bulk RNA changes may reflect shifts in trophoblast subtype composition rather than genuine transcriptional dysregulation within an individual cell type (127, 128). Critically, scRNA-seq applied specifically to preeclamptic placentas has demonstrated that ABCG1 is dramatically downregulated in the EVT compartment, while ABCA1 shows a divergent trend toward upregulation in the same EVT subpopulation. This cell type-specific subpopulation is directionally an opposite pattern that whole-tissue studies cannot detect (129). Spatial transcriptomics adds an orthogonal dimension by anchoring transcriptional data to anatomical coordinates within placental sections, enabling identification of transporter expression gradients across anchoring versus floating villi and correlating disease-related changes with spatial histopathology (130). Simultaneously, multi-omics integration is exposing pervasive post-transcriptional transporter regulation. The canonical example is ABCG2/BCRP, whose protein abundance increases substantially toward term despite stable mRNA (51). This is a discordance attributable to miRNA-mediated mechanisms detectable only through proteomics-transcriptomics integration. More broadly, multi-omics approaches applied to PE and IUGR placentas are beginning to link specific transporter protein abundance changes directly to downstream shifts in their substrate metabolomes, i.e., BAs, oxidized lipids, and cholesterol esters, converting correlative associations between transporter expression and disease into mechanistic evidence chains. The converging technologies, e.g., scRNA-seq, spatial transcriptomics, and multi-omics, define the methodological frontier of this field. The systematic application to the full human placental ABC transporter repertoire, across disease states and gestational stages. Taken together, the recent literature marks a convergence of structural precision, mechanistic depth, single-cell resolution, and translational ambition in the placental ABC transporter field, laying the groundwork for the regulatory and therapeutic insights discussed in the sections that follow.

**Table 4:** Summary of key recent studies advancing knowledge of ABC transporters in pregnancy complications.

Author (year)	Transporter(s)	Key findings	References
Alam & Locher (2023)	Representative human ABC transporters / human ABC transporter families	Comprehensive structural review using cryo-EM revealing molecular mechanisms of each subfamily; catalytic cycle insights via alternating-access model	(4)
Moore et al. (2023)	Broad: ABCA1, ABCB1, ABCG2, CFTR, others	21 ABC transporters linked to rare monogenic disorders; CFTR modulators proof-of-concept for drugging ABC transporters; therapeutic pipeline update	(7)
Liu et al. (2023)	ABCG1	ABCG1 polymorphisms in pregnant women with GDM; epigenetic regulation of ABCG1 expression; ABCG1 as epigenetic determinant of type 2 diabetes risk	(77)
Scott et al. (2022)	ABCB1, ABCG2 (P-gp, BCRP)	Preterm birth associates with increased placental MDR transporter expression, irrespective of pre-pregnancy BMI; MDR transporters as biomarkers for PTB	(65)
Sethuraman et al. (2022)	ABCA1, ABCG1, ABCG5, ABCG8	Dysregulation of cholesterol efflux ABC transporters during syncytialization in preeclampsia; ABCA1 modulates trophoblast fusion; potential PE biomarkers	(9)
Granitzer et al. (2022)	ABCC1 (MRP1)	MRP1 localization and function in human placenta; protective role against MeHg-induced oxidative stress; key regulator of GSH homeostasis at maternal-fetal interface	(69)
Fuenzalida et al. (2024)	ABCA1, ABCG1	Hypoxia-induced dysregulation of cholesterol homeostasis in preeclampsia; insights from human placental cells and tissues; cholesterol efflux-related ABC transporters impaired under hypoxia	(98)
Szatmári & Ducza (2023)	ABCB1 (P-gp), ABCG2 (BCRP)	Gestational age dependent expression changes in placental and intestinal P-gp and BCRP; drug therapy implications for GDM, infections, and antiemetics	(8)
Kotta-Loizou et al. (2024)	Multiple ABC transporters	Comprehensive review of drugs and chemicals transport across placental barrier; xenobiotic metabolism; *in vitro*, ex vivo, and in silico models for studying placental transfer	(126)

## 7 Transcriptional and post-transcriptional regulation of placental ABC transporters

The regulation of placental ABC transporters is a complicated process involving multiple levels of regulation depending on gestational age, oxygen levels, hormonal conditions, and inflammation state. Some examples of regulating factors are:

### 7.1 NRF2-Keap1 pathway

The nuclear factor erythroid 2-related factor 2 (NRF2) is an important transcriptional regulator of the antioxidant response and the transcriptional regulator of several placental ABC transporters such as BCRP, MRP1, MRP2, and P-gp. Under physiological conditions, NRF2 is constitutively ubiquitinated and degraded via its interaction with Keap1 (Kelch-like ECH-associated protein 1). Oxidative stress-induced modification of Keap1 cysteine residues liberates NRF2, allowing its nuclear translocation and transcriptional activation of antioxidant response element (ARE)-containing genes including ABCG2 and ABCC1. In preeclamptic placentas, NRF2 activity and downstream ABC transporter expression are significantly reduced, particularly in early-onset disease, while pharmacological NRF2 activation can restore transporter expression and partially reverse PE phenotypes in animal models (11).

### 7.2 HIF-1α/hypoxia signaling

Placental hypoxia—a hallmark of impaired spiral artery remodeling in preeclampsia and IUGR—activates HIF-1α, which paradoxically downregulates BCRP/ABCG2 through suppression of the aryl hydrocarbon receptor transcriptional pathway. Kozlosky et al. (1) demonstrated in BeWo trophoblast cells and high-altitude human placentas that HIF-1α activation reduces BCRP expression and function by 30–75%, increasing cellular retention of BCRP substrates (1). This creates a dangerous amplification loop: placental hypoxia reduces BCRP-mediated protection against oxidized lipids and xenobiotics precisely when oxidative toxin generation is highest (98).

### 7.3 Cytokine and growth factor regulation

Proinflammatory cytokines TNF-α and IL-1β—both elevated in preeclampsia and chorioamnionitis—significantly decrease BCRP mRNA and protein in primary trophoblasts, while epidermal growth factor and insulin-like growth factor II upregulate BCRP expression in parallel with promoting trophoblast differentiation. The above-mentioned data associate inflammation of complicated pregnancies with decreased barrier activity of the placenta through transporters (1, 8).

### 7.4 Gestational age dependent changes

Szatmári & Ducza (8) revealed that P-gp and BCRP possess gestational age dependent expression patterns in placenta and intestines. The authors reported an increase in BCRP protein expression without changes in mRNA in placentas approaching term; therefore, BCRP protein upregulation may be controlled by post-transcriptional mechanisms including miRNA and protein instability (8). P-gp protein expression in placenta was found relatively low during all gestational periods; however, maximum expression was registered in the first trimester. These results should be taken into account when planning safe drug administration in pregnant women since dosing of BCRP and P-gp substrate drugs (glyburide, antiretrovirals) will need to consider changes in gestational period (8, 54).

### 7.5 Autophagy-ABC transporter crosstalk

The study conducted by Shahnawaz et al. (78) introduced the connection between autophagy dysfunction and ABC transporter alteration in early miscarriage; dysfunction of autophagy causes accumulation of dysfunctional mitochondria with increased ROS and, thus, dysregulation of ABCG1 downregulation and ABCG2 upregulation in trophoblasts (78).

In a nutshell, the intricate regulatory networks governing placental ABC transporters underscore their critical role in fetal protection and maternal-fetal metabolic homeostasis. Future therapeutic strategies aiming to mitigate adverse pregnancy outcomes should prioritize the restoration of these protective barriers through the modulation of nuclear receptors and metabolic signaling pathways (131). Furthermore, utilizing advanced organoid models and microfluidic systems to study these pathways will be essential for assessing the placental permeability and fetal safety of novel therapeutic compounds (132, 133). Integrated approaches combining single-cell transcriptomics with these physiological models will further delineate how heterogeneous cell populations adapt their transporter profiles to the dynamic demands of gestation (134, 135). Continued research in this domain holds the potential to resolve long-standing inconsistencies regarding drug placental transport across species, facilitating more accurate, cost-effective, and safe medication strategies during pregnancy. These efforts are increasingly vital as the placental barrier faces emerging environmental challenges, such as the exposure to liquid crystal monomers that can dysregulate essential transplacental transport mechanisms.

## 8 Future perspectives

The structural advances enabled by cryo-EM, including the atlas of human ABC transporter crystallography by Alam and Locher hemic while (4), open new possibilities for rational drug design at the placental interface. The success of CFTR modulator therapy demonstrates that pharmacological correction of dysfunctional ABC transporters is achievable (7), though this analogy requires qualification. CFTR is not substantively expressed in trophoblasts, and ivacaftor-class modulators target CFTR-specific channel-gating and folding mechanisms that do not transfer to placental efflux transporters. More direct instructive evidence comes from human placental perfusion experiments and trophoblast cell studies. In ex vivo perfused human placentas, the P-gp/BCRP dual inhibitor elacridar increased maternal-to-fetal transfer of paclitaxel by approximately 50% and methadone by approximately 30%, while PSC833 augmented placental saquinavir transfer, confirming pharmacological accessibility at the human placental barrier (136, 137). The BCRP-specific inhibitor KO143 increased maternal-to-fetal transfer of the food carcinogen PhiP in the same perfusion system (138). The LXR agonist T0901317 upregulated ABCA1 in JAr trophoblast cells and first trimester placental explants under low-oxygen conditions modeling early-onset PE, establishing pharmacological proof-of-concept for restoring impaired trophoblast cholesterol efflux via nuclear receptor activation (139). MRP1 inhibition by MK-571 in BeWo trophoblast cells identified MRP1 as a pharmacologically tractable regulator of cAMP efflux in prostaglandin E2-stimulated trophoblasts, with relevance to preterm birth signaling (140). Most recently, trophoblast organoids derived from human villous tissue have been validated as a physiologically relevant ABC transporter drug transport platform in which P-gp, BCRP, MRP1, and MRP2 are correctly expressed, correctly localized, and selectively inhibited, providing the assay infrastructure needed for rational therapeutic screening (141). These findings collectively confirm pharmacological tractability while also illustrating the inherent risk: non-specific efflux inhibition can increase fetal drug exposure 2 to 4-fold, as shown by elevated fetal glyburide and nitrofurantoin concentrations in Bcrp-knockout pregnant mice (142). Therapeutic development at this interface therefore requires trophoblast-selective delivery mechanisms, gestational-stage-appropriate safety pharmacology, and mechanistically grounded target selection that distinguishes disease-adaptive upregulation from pathologically deleterious downregulation.

Five research priorities warrant focused effort. First, NRF2 activators such as bardoxolone and sulforaphane should be developed with trophoblast specificity to correct ABC transporter expression in PE. Second, gestational age-specific proteomic quantification of the full placental ABC transporter repertoire should be pursued using trophoblast organoid and placenta-on-a-chip platforms. Third, precision medicine approaches exploiting single nucleotide polymorphism data from ABCB4, ABCB11, ABCA1, and ABCG2 variants should be developed to predict pregnancy outcomes in ICP, PE, and GDM. Fourth, ABC transporter-informed drug delivery systems should be designed for fetal therapy applications. Fifth, the relationship between autophagy and ABC transporters during early trophoblast formation warrants investigation as a potential therapeutic target in recurrent miscarriage.

Three clinical translation priorities are actionable now without waiting for novel drug development. First, pharmacogenomics-guided prescribing: the ABCG2 Q141K variant reduces BCRP transport activity by approximately 50%, substantially increasing fetal drug exposure in carrier pregnancies (63). For glyburide, a first-line GDM treatment and high-affinity BCRP substrate, this translates to meaningfully higher fetal concentrations and clinically significant neonatal hypoglycemia risk. Routine ABCG2 Q141K genotyping in women initiating glyburide for GDM would permit a simple decision rule directing carriers toward insulin or a BCRP non-substrate alternative. ABCB4 heterozygous loss-of-function variants, present in up to 25% of ICP cases, similarly identify women at elevated ICP risk when exposed to drugs that impair canalicular phosphatidylcholine secretion, including synthetic estrogens (143). Second, drug-drug interaction awareness at the placental barrier: co-administration of ABCB1 or ABCG2 inhibitors with substrate drugs amplifies fetal drug exposure beyond what maternal plasma concentrations alone can predict. HIV-positive pregnant women on protease inhibitor-based antiretroviral regimens represent the highest-risk population for clinically significant placental drug interactions, and transporter profiling should inform antiretroviral selection in pregnancy (144). Third, transporter-informed drug selection: when intentional fetal drug delivery is required for conditions such as fetal arrhythmia or *in utero* cystic fibrosis treatment, selecting agents with low P-gp and BCRP affinity enhances placental transfer. When minimizing fetal exposure is the priority, preferring high-affinity efflux substrates deliberately exploits the placental barrier.

Realizing these applications requires a standardized, regularly updated ABC transporter substrate and inhibitor classification database for pregnancy-relevant drugs, a resource that does not yet exist in clinical-facing format, alongside integration of pharmacogenomics testing into standard antenatal care pathways. Advancing this framework would represent the most immediate translational dividend from two decades of placental ABC transporter research and would mark a meaningful shift from reactive toward predictive, preventive, and personalized maternal-fetal medicine.

## 9 Conclusions

ABC transporters function as primary gatekeepers of fetal chemical exposure and as active regulators of placental lipid metabolism, bile acid transport, and immune function. The evidence reviewed here supports the conclusion that ABC transporter dysfunction contributes mechanistically to obstetrical disease rather than arising purely as a secondary consequence. This relationship is most clearly established for ICP, where heterozygous loss-of-function variants in ABCB4 and ABCB11 are estimated to account for up to 20 to 25 percent of cases. In preeclampsia, gestational diabetes, IUGR, and preterm birth, transporter abnormalities more likely reflect a bidirectional pathobiology in which oxidative, hypoxic, and inflammatory stress suppresses transporter expression, which in turn amplifies downstream pathological consequences. These conditions share distinct ABC transporter dysregulation profiles tied to the NRF2, HIF-1α, TNF-α/IL-1β, and autophagy pathways.

Several limitations constrain the conclusions that can be drawn from current evidence. Human studies vary considerably in gestational age at sampling, placental biopsy site, and disease severity, which complicates cross-study comparisons. Many mechanistic insights derive from *in vitro* cell lines or animal models that do not fully reproduce human trophoblast architecture or transporter expression profiles (145). Placental ABC transporter expression is inherently gestational age dependent, yet most studies report findings at a single timepoint, making it difficult to distinguish disease-associated change from normal developmental variation (141). No approved therapeutic strategy currently targets a placental ABC transporter, and the safety margin for such interventions remains undefined. These gaps make longitudinal, multi-timepoint human studies and next-generation placental models necessary prerequisites before transporter-targeted therapy can be advanced responsibly toward the clinic.
